# 甘氨双唑钠对非小细胞肺癌放射治疗增敏作用的*Meta*分析

**DOI:** 10.3779/j.issn.1009-3419.2012.06.04

**Published:** 2012-06-20

**Authors:** 维维 任, 征 李, 登海 米, 克虎 杨, 金徽 田, 质钢 张

**Affiliations:** 1 730000 兰州，兰州大学循证医学中心 Evidence-based Medicine Center of Lanzhou University, Lanzhou 730000, China; 2 730000 兰州，兰州大学第一临床医学院 the First Clinical Medicine College of Lanzhou University, Lanzhou 730000, China; 3 730000 兰州，甘肃省第二人民医院 Gansu Provincial the Second People's Hospital, Lanzhou 730000, China

**Keywords:** 肺肿瘤, 放射治疗, 放射增敏剂, *Meta*分析, RCT, Lung neoplasms, Radiotherapy, Radiosensitizer, *Meta*-analysis, Randomized controlled trial

## Abstract

**背景与目的:**

甘氨双唑钠对非小细胞肺癌（non-small cell lung cancer, NSCLC）放射增敏作用的疗效和安全性目前并没有明确的证据，本研究系统评价甘氨双唑钠对NSCLC放射治疗增敏作用的临床疗效和安全性，为临床实践与更深入研究提供参考。

**方法:**

计算机检索Cochrane Library、PubMed、EMbase、中国生物医学文献数据库、中国学术期刊全文数据库、中国科技期刊数据库和数字化期刊全文数据库，同时辅助其它检索，收集所有关于甘氨双唑钠对NSCLC放射治疗增敏作用的随机对照试验（randomized controlled trial, RCT）。参考Cochrane质量评价标准进行质量评价，并利用RevMan 5.1软件进行统计学分析。

**结果:**

共纳入21个RCT，Meta分析结果显示，甘氨双唑钠联合放疗的近期疗效优于单纯放疗疗法（OR_合并_=3.29, 95%CI: 2.47-4.39, *P* < 0.000, 01）或优于放疗联合安慰剂（维生素C）疗法（OR_合并_=3.65, 95%CI: 2.25-5.92, *P* < 0.000, 01)；在生存质量提高率和1年、2年生存率方面两组差异均无统计学意义（*P*>0.05）；在放射性肺炎、放射性食管炎、血液学毒性、心脏毒性等安全指标方面两组差异均无统计学意义（*P*>0.05）。

**结论:**

甘氨双唑钠联合放疗疗法治疗NSCLC，近期疗效优于单纯放疗或放疗联合安慰剂（维生素C）疗法，并且不增加放疗不良反应，值得临床推广使用。

## Abstract (en)

**Background and objective:**

The efficacy and safety of radiosensitivity on non-small cell lung cancer (NSCLC) using metronidazole amino acidum natrium (CMNa) are yet to be proven. This study evaluates the efficacy and safety of radiosensitivity on NSCLC by CMNa to provide references for further clinical practice and research.

**Methods:**

Relevant randomized controlled trials (RCTs) were obtained from the Cochrane library, Pubmed, EMbase, CBM, CNKI, VIP, and Wan Fang databases. Dates were searched through other means. RCTs of radiosensitivity on NSCLC by CMNa were included. The data included in the study were evaluated and analyzed using the Cochrane Collaboration' s RevMan 5.1 software.

**Results:**

A total of 21 RCTs were included. The results of the meta-analyses showed that the total effective rate of the test group that received CMNa plus radiotherapy was higher than that of the control group that received radiotherapy alone (OR=3.29, 95%CI: 2.47-4.39, *P* < 0.000, 01) or radiotherapy plus placebo (OR=3.65, 95%CI: 2.25-5.92, *P* < 0.000, 01), respectively. No significant differences were found in the quality of life between one and two-year survival rates (*P*>0.05). No significant differences were found among radiation pneumonitis, radiation esophagitis, hematological toxicity, and cardiotoxicity (*P*>0.05).

**Conclusion:**

CMNa plus radiotherapy elicit beneficial effects in the treatment of NSCLC and produce fewer adverse effects. Therefore, this technique can be recommended and applied in clinics.

近年来肺癌的发病率和死亡率均呈逐年上升的趋势，已居癌症死亡首位。肺癌患者中约75%为非小细胞肺癌（non-small cell lung cancer, NSCLC），其中70%-80%因手术禁忌症或病灶不适合手术而失去了手术机会^[1]^。放射治疗仍是治疗恶性肿瘤的重要手段之一。放疗可以直接攻击肿瘤目标，具有直接和即时的优点。单纯放疗可使肿瘤周围的富氧部分敏感，但是乏氧的瘤体中心却抗拒射线，这也是放射治疗失败的主要原因^[2]^。英国科学家Adams等^[3]^发现硝基咪唑类化合物有亲电子特性，提出了著名的亲电子理论，使此类放射增敏剂的研究有了新的重大突破，但是由于不同程度的副作用，限制了其在临床中的使用。甘氨双唑钠（metronidazole amino acidum natrium, CMNa）是我国自行开发、研制的硝基咪唑类化合物，是一种新型放射增敏剂，与具有亲水性和亲肿瘤细胞的化学结构连接形成一个桥式结构而提高增敏活性，同时解决了硝基咪唑类化合物毒副作用大、不能用于临床的一大难题^[4]^。目前虽然有甘氨双唑钠对NSCLC放射治疗增敏作用的临床试验报道，但尚无相应的系统评价做指导。本文旨在评价甘氨双唑钠对NSCLC放射治疗增敏作用的疗效和安全性，为以后的临床研究和临床治疗决策提供参考依据。

## 1 材料与方法

### 1.1 文献纳入标准

#### 1.1.1 研究类型

随机对照试验（randomized controlled trial, RCT），无论是否采用分配隐藏或盲法。

#### 1.1.2 研究对象

① 经病理组织学或细胞学确诊的NSCLC患者；②肺原发肿瘤患者；③除放射治疗以外未接受任何治疗；④种族、国籍、年龄、性别不限。

#### 1.1.3 干预措施

放疗联合甘氨双唑钠（实验组）对比放疗或放疗联合安慰剂（维生素C）（对照组）。

#### 1.1.4 测量指标

① 近期疗效：完全缓解（complete response, CR），部分缓解（partial response, PR），总有效率（overall response rate, ORR）；②生存率（survival rate）；③不良反应发生率（rate of adverse reaction）；④卡氏评分（Karnofsky, KPS）提高率。

### 1.2 检索方法与策略

计算机检索Cochrane Library、PubMed、EMbase、中国生物医学文献数据库、中国学术期刊全文数据库、中国科技期刊数据库和数字化期刊全文数据库，检索时限为自各数据库建库至2011年12月1日。以“甘氨双唑钠OR希美钠（Metronidazole Amino Acidum Natrium OR Sodium Glycididazole for Injection OR CMNa, #1）”和“肺癌OR非小细胞肺癌（lung cancer OR non-small cell lung cancer OR non -small cell lung carcinoma OR non-small cell lung neoplasms OR NSCLC, #2）”作为中英文检索词进行主题词和自由词检索，然后从“#1 AND #2”的检索结果中筛选出所有的RCT。接着对相关文献以及参考文献进行手检，并结合Google Scholar、Medical Martix等搜索引擎在互联网上查找相关文献。最后与本领域的专家、相关文献的通讯作者联系以获取以上检索未发现的信息。

### 1.3 文献筛选和质量评价

#### 1.3.1 文献筛选和提取资料

由2位评价者（李征和任维维）独立阅读检索所得文献的题目和摘要，在排除明显不符合纳入标准的文献后阅读可能符合纳入标准的文献全文，交叉核对纳入文献的结果。对是否纳入而有分歧的文献通过讨论的方式解决，并由第3位评价者（米登海）决定其是否纳入。对最终纳入文献进行资料提取，提取的内容包括入选标准、样本量、研究的条件、干预的内容、测量指标、随访时间、失访率、失访原因、统计学方法、研究对象的基本资料、抽样和分组的方法。对缺少的资料通过电话或邮件与作者联系进行补充。在涉及含有多组研究的RCT时提取与本文相关的实验组与对照组数据。

#### 1.3.2 文献质量评价

参考Cochrane协作网提供的标准并结合本研究特点进行质量评价：①具体的随机分配方法；②是否进行分配方案隐藏；③结局指标的基线情况是否可比；④是否报告失访情况；⑤如有失访，是否采用意向性分析（intention-to-treat analysis, ITT）来检验结论的稳健性。本文不以盲法作为评价标准，因为纳入研究的测量结果均为客观指标，盲法对研究结果的影响较小。如果纳入的研究满足以上5条质量评价标准，则该研究存在偏倚的可能性最小；如果完全不满足5条质量评价标准，则不能排除该研究存在偏倚的可能性。

### 1.4 统计方法

采用Cochrane协作网提供的RevMan 5.1软件进行*Meta*分析。首先分析纳入研究的临床异质性，利用*I*^2^和*P*确定纳入研究间的统计学异质性，其临界值分别设定为*P*=0.1、*I*^2^=50%，如果*P*>0.1、*I*^2^ < 50%，说明各研究间存在统计学同质性，采用固定效应模型进行结果分析；如果*P* < 0.1、*I*^2^ > 50%，说明各研究间存在统计学异质性，分析其异质性来源，根据可能导致异质性的因素进行亚组分析，对仍无法消除统计学异质性但从临床意义上看可以合并的文献用随机效应模型分析；如果两组间异质性过大，则采用描述性分析。最终各效应量的统计结果以优势比（odds ratio, OR）的95%的可信区间（confidence interval, CI）表示。

### 1.5 发表偏倚

以各研究近期有效率的OR值为横坐标，以标准差（standard error, SE）的logOR值为纵坐标，利用RevMan 5.1软件描绘出漏斗图。通过观察数据点分布的对称性来评价是否可能存在发表偏倚。

## 2 结果

### 2.1 文献的检索及筛选结果

初次检索获得相关文献235篇，经阅读文题、摘要和全文后排除未设立对照的临床总结、非RCT、非临床研究、干预措施不符、基线可比性差、研究对象不符、有其它肿瘤患者而不能独立提取肺癌数据以及重复数据资料的研究，最终21篇研究文献符合系统评价纳入标准。文献的筛选流程见图 1。

**1:**
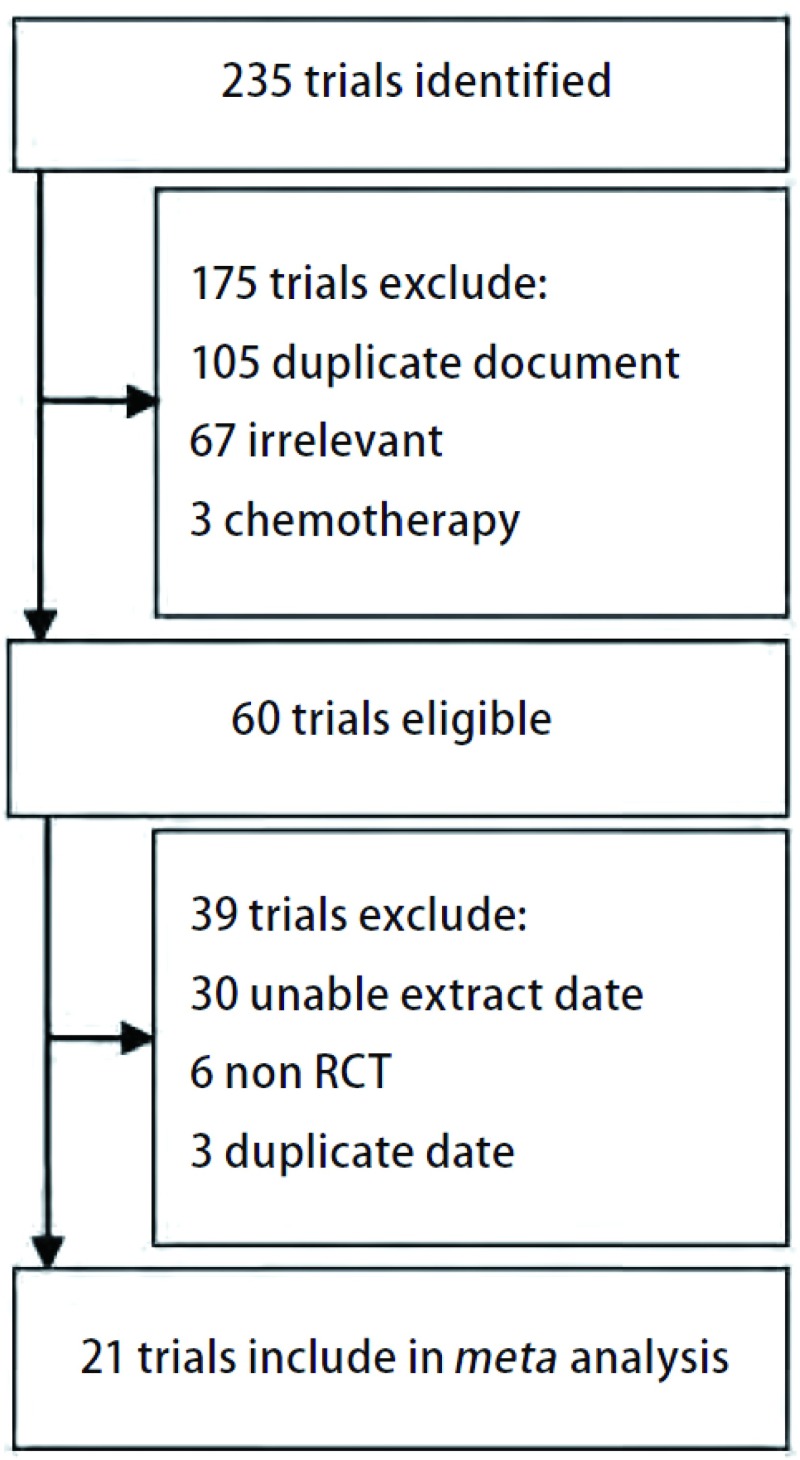
纳入研究筛选流程图 Selection of trials. RCT: randomized controlled trial.

### 2.2 纳入研究的基本特征

共纳入21项研究^[5-25]^。实验组和对照组患者KPS > 70，年龄≤80岁，两组基线资料具可比性。其中15项研究^[5, 8-11, 14-19, 21, 23-25]^采用甘氨双唑钠联合放疗对比单纯放疗疗法，6项研究^[6, 7, 12, 13, 20, 22]^采用甘氨双唑钠联合放疗对比放疗联合安慰剂或维生素C疗法。实验组甘氨双唑钠的剂量为800 mg/m^2^，用100 mL生理盐水稀释溶解，并在30 min内完成静脉滴注，患者无不良反应后于60 min内进行放疗。近期疗效的测定均按照世界卫生组织实体瘤疗效判定标准进行。纳入研究的基本特征见表 1。

**1:** 纳入临床研究的一般资料 The characteristics of included studies

Included studies	Cases	KPS scores	Clinicopathological staging	Intervention		Outcome
				Treatment	Control	
Li HX 2007^[5]^	86	> 70	Ⅲ	3DCRT plus CMNa	3DCRT	ABCDEGHIJ
Zhao QW 2007^[6]^	48	≥70	Ⅲ	CTR plus CMNa	CTR plus vitamin C	ABCIJKL
Li ZW 2007^[7]^	56	≥70	Ⅱ, Ⅲ	CTR plus CMNa	CTR plus vitamin C	ABC
Zhang XH 2009^[8]^	32	≥70	Ⅲ	3DCRT plus CMNa	3DCRT	ABCGH
Deng YN 2009^[9]^	152	≥70	Ⅲ	CTR plus CMNa	CTR	ABC
Ji N 2010^[10]^	90	≥70	Ⅲ	CTR plus CMNa	CTR	ABCGHMN
Qian YH 2007^[11]^	98	≥70	Ⅱ, Ⅲ	CTR plus CMNa	CTR	ABCJ
Guo YX 2007^[12]^	48	≥70	Ⅰ, Ⅱ	3DCRT plus CMNa	3DCRT plus vitamin C	ABC
Dang YZ 2006^[13]^	72	≥70	Ⅱ, Ⅲ	3DCRT plus CMNa	3DCRT plus vitamin C	ABC
Zhang N 2008^[14]^	80	≥70	Ⅱ, Ⅲ	CTR plus CMNa	CTR	ABC
Han JQ 2004^[15]^	402	≥70	Ⅰ, Ⅱ, Ⅲ	CTR plus CMNa	CTR	ABCO
Liu YC 2007^[16]^	65	Unclear	Ⅲ	CTR plus CMNa	CTR	ABCDEGHM
Liu JM 2010^[17]^	46	≥70	Ⅲ	CTR plus CMNa	CTR	ABCJIKL
Jia JH 2008^[18]^	64	≥70	Ⅲ	3DCRT plus CMNa	3DCRT	ABCHMP
An YM 2008^[19]^	120	> 60	Ⅲ, Ⅳ	SR plus CMNa	SR	ABCDEF
Chen MC 2009^[20]^	60	> 70	Ⅱ, Ⅲ	3DCRT plus CMNa	3DCRT plus vitamin C	ABCDEF
Chen RX 2006^[21]^	42	≥70	Ⅲ	3DCRT plus CMNa	3DCRT	ABCGH
Wang TJ 2008^[22]^	120	≥60	Ⅲ	3DCRT plus CMNa	3DCRT plus placebo	ABC（G）IJKLNQ
Wang JC 2009^[23]^	77	> 70	Ⅲ	3DCRT plus CMNa	3DCRT	ABCGHIM
Gao HF 2011^[24]^	56	≥70	Ⅱ, Ⅲ	IMRT plus CMNa	IMRT	ABC
Song X 2006^[25]^	80	Unclear	Ⅲ	CTR plus CMNa	CTR	ABCGHMNQRS
A: CR; B: PR; C: CR+PR; D: 1-year survival rate; E: 2-year survival rate; F: 3-year survival rate; G: radiation pneumonitis; H: radiation esophagitis; I: heart toxicity; J: hematologic toxicity; K: kidney toxicity; L: liver toxicity; M: myelosuppression; N: radiodermatitis; O: anaphylaxis; P: nausea and vomiting; Q: weight loss; R: blood biochemistry; S: nerve toxicity; (G): the two outcomes of radiation pneumonitis; 3-DCRT: 3-Dimension conformal radiation therapy; CTR: conventional radiotherapy; SR: stereotactic radiotherapy; IMRT: intensity modulation radiation therapy. CR: complete response; PR: partial response.						

### 2.3 文献质量评价

各研究组间因素基本匹配，具有可比性。所有纳入研究均声明随机分组，4项研究^[8, 17, 21, 24]^进行随机数字法分组，2项研究^[5, 22]^描述了信封法分配隐藏，3项研究^[5, 7, 16]^报道了失访。因此21项研究均有发生偏倚的中度可能性。各纳入研究的质量评价结果详见表 2。

**2:** 纳入研究方法学质量评价 Quality assessment of included studies

Included studies	Randomization	Allocated concealment	Baseline	Lost of follow-up	ITT analysis
Li HX 2007^[5]^	Unclear	Envelope	Compatibility	Yes（*n*=6）	No
Zhao QW 2007^[6]^	Unclear	Unclear	Compatibility	No	No
Li ZW 2007^[7]^	Unclear	Unclear	Compatibility	Yes（*n*=1）	No
Zhang XH 2009^[8]^	Random digits	Unclear	Compatibility	Unclear	No
Deng YN 2009^[9]^	Unclear	Unclear	Compatibility	Unclear	No
Ji N 2010^[10]^	Unclear	Unclear	Compatibility	Unclear	No
Qian YH 2007^[11]^	Unclear	Unclear	Compatibility	Unclear	No
Guo YX 2007^[12]^	Unclear	Unclear	Compatibility	Unclear	No
Dang YZ 2006^[13]^	Unclear	Unclear	Compatibility	Unclear	No
Zhang N 2008^[14]^	Unclear	Unclear	Compatibility	Unclear	No
Han JQ 2004^[15]^	Unclear	Unclear	Compatibility	Unclear	No
Liu YC 2007^[16]^	Unclear	Unclear	Compatibility	Yes（*n*=2）	No
Liu JM 2010^[17]^	Random digits	Unclear	Compatibility	Unclear	No
Jia JH 2008^[18]^	Admission order random	Unclear	Compatibility	Unclear	No
An YM 2008^[19]^	Unclear	Unclear	Compatibility	Unclear	No
Chen MC 2009^[20]^	Unclear	Unclear	Compatibility	Unclear	No
Chen RX 2006^[21]^	Random digits	Unclear	Compatibility	Unclear	No
Wang TJ 2008^[22]^	Unclear	Envelope	Compatibility	No	No
Wang JC 2009^[23]^	Unclear	Unclear	Compatibility	Unclear	No
Gao HF 2011^[24]^	Random digits	Unclear	Compatibility	Unclear	No
Song X 2006^[25]^	Unclear	Unclear	Compatibility	Unclear	No
ITT: intention to treat					

### 2.4 *Meta*分析结果

#### 2.4.1 近期疗效

除章霓等^[14]^和宋欣等^[25]^2项研究只比较了CR（两组差异有统计学意义，*P* < 0.05），其余19项研究^[5-13, 15-24]^比较了CR+PR，以对照组的干预方式不同分成甘氨双唑钠联合放疗对比单纯放疗（13项研究^[5, 8-11, 15-20, 23, 24]^）和甘氨双唑钠联合放疗对比放疗联合安慰剂（维生素C）（6项研究^[6, 7, 12, 13, 20, 22]^）两个亚组。经过系统分析显示两亚组内具有临床同质性和统计学同质性（*I*^2^=0%, *P*=0.99; *I*^2^=0%, *P*=0.94），因此亚组内可以合并结果，采用固定效应模型进行*Meta*分析（图 2），结果显示差异均具有统计学意义（OR_合并_=3.29, 95%CI: 2.47-4.39, *P* < 0.000, 01）和（OR_合并_=3.65, 95%CI: 2.25-5.92, *P* < 0.000, 01)。

**2:**
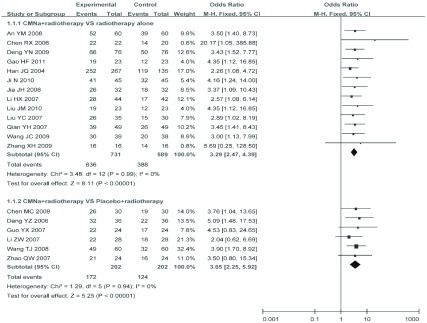
有效率的*meta*分析 *Meta*-analysis of the included trials on effective rate

#### 2.4.2 KPS评分提高率

有2项研究^[9, 11]^分别统计了甘氨双唑钠联合放疗对比单纯放疗KPS评分的提高例数，其中邓研农^[9]^的研究显示差异有统计学意义（OR_合并_=3.33, 95%CI: 1.68-6.63, *P* < 0.001）；而钱永红^[11]^的研究显示差异无统计学意义（OR=1.10, 95%CI: 0.47-2.53, *P*>0.05）。

#### 2.4.3 1年、2年生存率

有4项研究^[5, 16, 19, 20]^分别统计了甘氨双唑钠联合放疗对比单纯放疗的1年、2年生存率。经过异质性检验显示具有临床同质性和统计学同质性（*P*=0.81, *I*^2^=0; *P*=0. 94, *I*^2^=0），采用固定效应模型进行*Meta*分析（图 3），结果显示差异均无统计学意义（OR_合并_=1.49, 95%CI: 0.92-2.43, *P*=0.11）和（OR_合并_=1.37, 95%CI: 0.76-2.45, *P*=0.30)。

**3:**
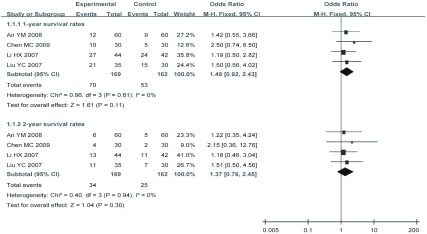
1年, 2年生存率的*meta*分析 *Meta*-analysis of the 1-, 2-year survival rate

#### 2.4.4 安全性

王铁君等^[22]^的研究对放射性肺炎做了两项不同的数据统计，估计其中一项为放射性食管炎的数据，应该是书写错误，设法与作者联系但最终未获得信息，对这两项数据均不纳入分析。其余纳入研究的*Meta*分析结果显示，甘氨双唑钠联合放疗与单纯放疗或放疗联合安慰剂（维生素C）在放射性肺炎、放射性食管炎、血液学毒性、心脏毒性等安全指标方面差异均无统计学意义（*P*>0.05）。见表 3。

**3:** 甘氨双唑钠联合放疗与单纯放疗或放疗联合安慰剂（维生素C）安全性比较的*Meta*分析 *Meta*-analysis of safety for CMNa plus radiotherapy

Adverse effects	Included studies	Treatment			Control			Heterogeneity	
		*n*	*N*		*n*	*N*		*I*^2^	*P*
Radiation esophagitis	8^[5, 8, 10, 16, 18, 21, 23, 25]^	119	273		120	254		0	0.99
Radiation pneumonitis	7^[5, 8, 10, 16, 21, 23, 25]^	78	241		76	231		0	0.99
Hematologic toxicit（myelosuppression）	7^[5, 10, 16, 18, 21, 23, 25]^	63	284		59	276		0	0.95
Radiodermatitis	3^[10, 22, 25]^	80	145		75	145		0	0.90
Weight loss	2^[22, 25]^	58	100		60	100		-	-
Liver and kidney	2^[6, 22]^	6	84		10	84		0	0.41
Heart	4^[5, 6, 22, 23]^	18	167		19	164		0	0.76
Nausea and vomiting	1^[18]^	4	32		4	32		-	-
CMNa: metronidazole amino acidum natrium									

#### 2.4.5 文章发表偏倚

对纳入文献进行漏斗图（图 4）分析，19项研究^[5-13, 15-24]^的散点分布不对称，提示纳入分析的研究可能存在发表偏倚。

**4:**
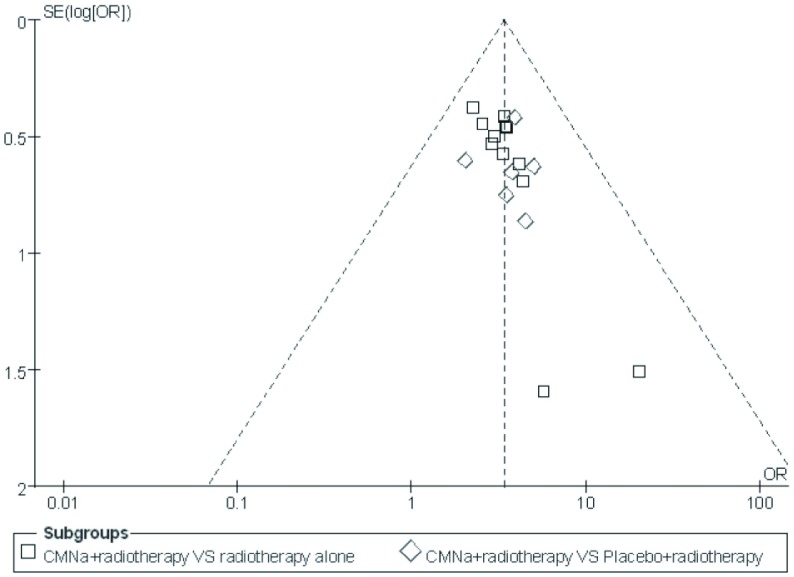
纳入研究的漏斗图 Funnel plot for the response rate of included trials

## 3 讨论

肺癌已成为目前人类癌症死亡的主要原因，临床上约86%的肺癌患者在确诊时已属晚期。长期以来放疗是治疗中晚期肺癌特别是NSCLC的主要手段，但是根治性放疗后大部分患者在1年-2年内仍死于局部复发和远处转移^[26]^。肺癌放疗疗效差的一个重要原因是肿瘤组织中存在乏氧细胞，这些乏氧细胞虽然暂时不能分裂，但仍保持增殖能力，一旦乏氧状态得到改善，就能够继续分裂增殖，最终使肿瘤复发和转移。无论在放疗过程中吸纯氧还是高压氧疗，由于肿瘤血管的异常以及栓塞的形成，氧不易扩散到乏氧细胞区而使放疗疗效较差。

甘氨双唑钠是一种新型硝基咪唑类化合物^[27]^，放疗增敏作用机理包括：①射线作用于肿瘤细胞后引起细胞分子损伤，并且甘氨双唑钠亲电子作用能够转移肿瘤细胞受损的电子，使损伤固定下来，从而明显增强放疗效果；②甘氨双唑钠对DNA修复酶特别是聚合酶β有抑制作用，从而抑制肿瘤细胞中受损DNA分子的修复以及肿瘤细胞特别是乏氧细胞的潜在致死损伤修复和亚致死损伤修复，进而提高放疗对肿瘤细胞的杀灭作用。

本系统评价结果显示，在近期疗效方面甘氨双唑钠联合放疗疗法优于单纯放疗和放疗联合安慰剂（维生素C）疗法；生存质量方面邓研农^[9]^报道甘氨双唑钠联合放疗优于单纯放疗，而钱永红^[11]^报道甘氨双唑钠联合放疗与单纯放疗相比无明显差异，由于纳入的研究中只有这两项研究统计了KPS评分提高的例数，目前尚不能对甘氨双唑钠联合放疗疗法在改善NSCLC患者的生存质量方面得出非常肯定的结论；1年、2年生存率方面，4项研究^[5, 16, 19, 20]^报道了甘氨双唑钠联合放疗与单纯放疗或放疗联合安慰剂（维生素C）相比无明显差异，鉴于只纳入了含有331例患者的4项研究而可能存在统计学效能问题，因此从这些小样本量的研究得出的结论需要谨慎对待，当有不同结论的新研究发表时这个结论可能易于改变；安全性方面甘氨双唑钠联合放疗疗法并不增加放射性食管炎、放射性肺炎、血液学毒性、心脏毒性等不良反应的发生率，但对于体重减轻、肝肾功能异常、恶心呕吐等不良反应，由于纳入研究的数量有限，需要更多相关的临床研究加以验证。

影响本次系统评价结果论证强度的因素可能有：①本研究纳入的21项RCT^[5-25]^分配隐藏评价不充分，虽然3项研究^[5, 7, 16]^报道了失访情况，但都未进行ITT分析，并且大部分RCT未描述具体随机方法，因此选择偏倚的可能性不能排除；②放疗方案的不同（三维适形放疗、常规放疗、调强放疗）可能会对结果造成一定的影响；③ *Meta*分析漏斗图不对称，提示各研究可能存在发表偏倚。为了保证文献搜集的广泛性，文献检索过程中使检索策略达到了足够的敏感性，尽量减少发表偏倚对系统评价结果的影响，但可能因阴性结果的研究未能公开发表而造成发表偏倚仍无法避免。

综上，甘氨双唑钠联合放疗疗法治疗NSCLC，近期疗效优于单纯放疗或放疗联合安慰剂（维生素C）疗法，并且不增加放疗的不良反应。但对于肿瘤患者生存率和生存质量也显得尤为重要，今后需要更多的RCT对患者的生存率和生存质量进行进一步研究。
